# Malaria transmission blocking activity of *Anopheles stephensi* alanyl aminopeptidase N antigen formulated with MPL, CpG, and QS21 adjuvants

**DOI:** 10.1371/journal.pone.0306664

**Published:** 2024-07-05

**Authors:** Zeinab Pourhashem, Leila Nourani, Sakineh Pirahmadi, Hemn Yousefi, Jafar J. Sani, Abbasali Raz, Sedigheh Zakeri, Navid Dinparast Djadid, Akram Abouie Mehrizi

**Affiliations:** Pasteur Institute of Iran, Malaria and Vector Research Group (MVRG), Biotechnology Research Center (BRC), Tehran, Iran; Ehime Daigaku, JAPAN

## Abstract

**Backgrounds:**

Malaria, a preventive and treatable disease, is still responsible for annual deaths reported in most tropical regions, principally in sub-Saharan Africa. Subunit recombinant transmission-blocking vaccines (TBVs) have been proposed as promising vaccines to succeed in malaria elimination and eradication. Here, a provisional study was designed to assess the immunogenicity and functional activity of alanyl aminopeptidase N (APN1) of *Anopheles stephensi*, as a TBV candidate, administered with MPL, CpG, and QS21 adjuvants in the murine model.

**Methodology/Principal findings:**

The mouse groups were immunized with recombinant APN1 (rAPN1) alone or formulated with CpG, MPL, QS-21, or a combination of adjuvants (CMQ), and the elicited immune responses were evaluated after the third immunization. The standard membrane feeding assay (SMFA) measured the functional activity of antibodies against bacterial-expressed APN1 protein in adjuvanted vaccine groups on transmission of *P*. *falciparum* (NF54) to *An*. *stephensi* mosquitoes. Evaluation of mice vaccinated with rAPN1 formulated with distinct adjuvants manifested a significant increase in the high-avidity level of anti-APN1 IgG and IgG subclasses; however, rAPN1 induced the highest level of high-avidity anti-APN1 IgG1, IgG2a, and IgG2b antibodies in the immunized vaccine group 5 (APN1/CMQ). In addition, vaccine group 5 (receiving APN1/CMQ), had still the highest level of anti-APN1 IgG antibodies relative to other immunized groups after six months, on day 180. The SMFA data indicates a trend towards higher transmission-reducing activity in groups 2 and 5, which received the antigen formulated with CpG or a combination of three adjuvants.

**Conclusions/Significance:**

The results have shown the capability of admixture to stimulate high-affinity and long-lasting antibodies against the target antigen to hinder *Plasmodium* parasite development in the mid-gut of *An*. *stephensi*. The attained results authenticated APN1/CMQ and APN1/CpG as a potent APN1-based TBV formulation which will be helpful in designing a vaccine in the future.

## Background

Malaria, a preventive and treatable disease, is still responsible for more than 409000 annual deaths reported in most tropical regions (WHO, 2022). With an estimation of two-thirds of deaths among children under five, malaria remains an important cause of child mortality in many settings, principally in sub-Saharan Africa. Regardless of the reduction in the total number of malaria cases and deaths since 2000, as a result of the COVID-19 pandemic and limitations in essential malaria services, about 229 million cases and 627000 deaths have been reported in malaria-endemic countries [1,2]. Despite various tools being used to combat malaria [3,4], malaria still poses a significant burden on public health [5]. Resistance to available anti-malarial drugs and insecticides necessitates the inclusion of more efficacious vaccines to combat malaria [5]. The goal of eliminating and eventually eradicating malaria will be achieved by inhibition of transmission to the *Anopheles* mosquito and reducing the parasite reservoirs. According to the PATH Malaria Vaccine Initiative Portfolio [http://www.malariavaccine.org/rd-portfolio.php] [5], transmission-blocking vaccines (TBVs), by aiming different antigens associated with the sexual stage of *Plasmodium* life cycle, zygotes, gametes, and ookinetes and antigens from mosquito stages are considered as indispensable tools to meet this ambitious goal.

The number of recently discovered candidate antigens for this type of vaccine has increasingly grown by various studies. Among the promising TBV antigens, *Anopheles* Anopheline alanyl aminopeptidase N (AnAPN1), a ligand for *Plasmodium* ookinetes, has shown the potency to interrupt the sporogonic life cycle of *Plasmodium* species [6]. Full-length AnAPN1 is a 1020 amino acids residue that has been predicted to be a 113.5 kDa protein [7], and antibodies against the N terminal of mature AnAPN1^60–195^ (~18 kDa) have shown the potency to interrupt the sporogonic life cycle of *Plasmodium* species [6–8]. Notably, Atkinson et al., (2015) demonstrated that Peptide 1 (aa: 60–74) and 5 (aa: 95–109) in the N-terminal (aa 60–195) were immunogenic decoy epitopes [7]. More recently, Bender et al., (2021) reported on the 2nd generation of the APN1 vaccine against *An*. *gambiae* includes transmission-blocking epitopes (Peptide 7, aa: 98–123 and 9, aa: 173–194), but not the Peptide 1 region. However, there is no data for the homologous protein in *An*. *stephensi;* hence, our study aims to revisit the entire N-term APN1 protein in *An*. *stephensi* [9].

In our laboratory, *An*. *stephensi apn1* gene characterization was performed by rapid amplification of cDNA ends (3ʹ -RACE), genome walking approaches, and the *An*. *stephensi* APN1 protein (AsAPN1) was expressed in insect cell line Sf9 via the *baculovirus* expression platform. *In silico* analysis revealed similar structural features and immunological properties of AsAPN1 and *An*. *gambiae* APN1 (AgAPN1) Results from the previous study performed in our laboratory confirmed that the whole AsAPN1 protein and the N-terminal formulated in Freund’s adjuvant have the same Transmission-Reducing Activity (TRA). Therefore, our study aims to investigate the entire N-term APN1 protein (containing peptides 1, 5, 7, and 9) in *An*. *stephensi*, as a transmission-blocking vaccine, using different adjuvant formulations.

Humoral responses and IgG antibodies against TBV antigens intervene in ookinete binding and interrupt the sporogonic cycle within the vector. [10]. This process, along with the activation of effector CD4+ and/or CD8+ T cells by both humoral and cellular immune responses, stimulates cytokine secretion, leading to the production of high-affinity antibodies and the development of memory B cells. A well-formulated vaccine will stimulate specific immune responses of various types with minimal toxicity, enhancing the durability, quality, and effectiveness of the immune reactions elicited [11,12].

To improve the immune responses, the use of adjuvants in vaccine formulations is suggested due to the poor immunogenicity of recombinant antigens. Adjuvants play a vital role in eliciting a robust and enduring immune response against the antigen. One of the human-compatible adjuvants is CpG ODNs, which contain non-methylated CpG motifs of guanidine cytosine phosphate and have been recognized as TLR-9 agonists [13] and potent *in vitro* B-cell activators [14]. DNA adjuvant CpG has been utilized for various disease vaccines that approve the efficiency of adjuvanted boost immunizations, H7N9 avian influenza A virus [15], *Toxoplasma gondii* virus-like particle vaccine (VLP) [16], *P*. *falciparum* [17–19], and cancer [20]. Another potent TLR adjuvant is Monophosphoryl lipid A (MPL), a lipopolysaccharide (LPS) derivative that activates the immune system by interacting with TLR4 [21]. This adjuvant can promote both humoral and cellular responses [22]. MPL adjuvant has been applied in some vaccines, including a nano-liposomal vaccine for breast cancer [23], influenza [24,25], and malaria [17,19,26]. QS21 is a saponin-based component with significant adjuvant activity and low toxicity in model organisms. This compound has the aptitude to stimulate cellular and humoral immunity, particularly IgG2a isotype [11,27].

The synergistic effect of these adjuvants has shown promising results in amplifying the immune response and improving vaccine efficacy. However, the response can vary based on the antigen’s characteristics, including size, structure, and immunogenicity. While adjuvant combinations like CpG, MPL, and QS21 have demonstrated significant potential in augmenting the immune response [4], the specific outcome may not always be consistent across different antigens. Therefore, it is crucial to carefully select the appropriate adjuvant combination for each antigen and disease to optimize vaccine efficacy [28].

This study hypothesizes that combining the N-terminal region of APN1 antigen with human-compatible adjuvant compounds (CpG, MPL, and QS-21) would increase the immunogenicity of the antigen as well as the avidity and functional transmission-reducing activity (TRA) of anti-APN1 antibodies in comparison with non-adjuvanted immunized BALB/c mice. In this regard, the intimate assessment of humoral and cellular immune responses in immunized mouse groups was evaluated to investigate IgG antibody titer, isotype profiling, avidity, and persistence of responses. The effectiveness of this approach was determined by analysis of the inhibitory potency of oocyst formation by anti-APN1 antibodies induced in different immunized mice groups. The obtained results from this study will be helpful in designing an APN1-based TBV in the future.

## Material and methods

### Cloning, expression and purification of APN1 protein

At first, the N-terminal region of AsAPN1 and AgAPN1 were aligned and the peptides 1,4, 5, 7, and 9 that are the targets of inhibitory antibodies were compared (S1 Fig), which showed variation in the peptides between antigens from *An*. *stephensi* and *An*. *gambiae*. Therefore, to assess the AsAPN1 as an *An*. *stephensi*-specific TBV candidate, this gene was cloned and expressed. Initially, the genomic DNA of *An*. *stephensi mysorensis* mosquitoes (maintained at the National Insectarium of the Pasteur Institute of Iran) were extracted by the DNA extraction kit (Favorgene, Taiwan). Specific primers for the amplification of N-terminal fragment (aa: 58–196; nt: 417 bp) of *An*. *stephensi* APN1 (GenBank accession no. MF143582.1) was designed considering the *NdeI* and *XhoI* enzyme cleavage sites in the 5’ ends of forward and reverse primers, respectively. The amplified N-terminal APN1 region was cloned between *XhoI-NdeI* sites in pET23a plasmid (accession no. OQ096831) and then transformed to the prokaryotic expression host, *E*. *coli* BL21(DE3), for expressing the recombinant protein. The obtained recombinant plasmid, pET23a-APN1, was confirmed by the colony PCR, restriction-enzyme analysis using *NdeI* and *XhoI* enzymes, and sequencing. Transformants *E*. *coli* BL21(DE3) harboring pET23a-APN1 were cultured in LB medium at 37°C and induced by 1mM isopropyl-β-D-thiogalactopyranoside (IPTG; Thermo Scientific, USA) at the turbidity absorbance of the medium λ600nm = 0.8 (OD). For the purification of recombinant APN1, the cells were harvested 16h after induction, and bacterial pellet was suspended in the lysis buffer (Tris-HCl 20 mM, Urea 8 M, NaCl 500 mM, and Imidazole 20 mM, pH 7.5), and consequently was incubated at 4°C for 90 min. The suspension was sonicated 20 pulses at 70-s intervals and 75% amplitude in 5 cycles (Ultraschallprozessor, Deutschland, Germany), then centrifuged at 7000xg for 15 min. The supernatant was collected and incubated with the Ni–NTA agarose (Qiagen, Hilden, Germany) at 4°C for 90 min. The resin was packed into a column after washing the un-bound proteins with a 10-column volume of wash buffer (Tris-HCl 20 mM, Urea 4M, NaCl 750 mM, and Imidazole 40 mM, pH 7.5), and the bound proteins to Ni-NTA were eluted using elution buffer (Tris-HCl 20 mM, Urea 2M, NaCl 500 mM and Imidazole 250 mM, pH 7.2). The purified protein was desalted using Econo-Pac 10 DG columns (Bio-Rad, Hercules, CA, USA) according to the manual described by the manufacturer. The purified APN1 was analyzed by 12% sodium dodecyl sulfate-polyacrylamide gel electrophoresis (SDS-PAGE) and subsequently was confirmed by the Western blot analysis using monoclonal penta-His antibody (Qiagen). The concentration of recombinant APN1 (rAPN1) was measured by calorimetric Bradford assay. Moreover, *E*. *coli* endotoxin level was measured using the LAL kit (PYROSTAR^TM^ ES-F, FUJIFILM, USA) by mixing 0.2 mL of sample in a test tube, along with standard dilutions and incubating using a block heater at 37 ± 1°C, 60 ± 2 minutes, without subjecting to vibration and checking for gel clot after completion of heating.

### Immunization of BALB⁄c mice with rAPN1 in different formulations

Mouse immunization was performed for inbred female BALB/c mice obtained from the Department of Laboratory Animal Science at the Pasteur Institute of Iran (Karaj), which were kept in the animal house for one week for adaptation to the new conditions. The animal procedures were approved by the Committee of Animal Ethics of the Pasteur Institute of Iran (IR.PII.REC.1399.103).

The optimized concentration of antigen for immunization was achieved in the pre-test before immunizing the main mouse groups. For this purpose, different antigen concentrations were examined in several groups. Briefly, the mice (n = 18) were divided into six groups and immunized with 5, 10, or 20 μg of rAPN1 with CFA/IFA adjuvant (groups 1–3) and without adjuvant (groups 4–6) at the base of the tail, three times at two-week intervals. Subsequently, the sera were collected on days 10, 24, and 38 after the first immunization and analyzed for the antibody responses. In addition, cytokines (IFN-γ and TNF) were measured after the stimulation of splenocytes of immunized mice with rAPN1 (data in S1 File). For the main test, inbred female BALB⁄c mice (6- to 8-week-old) (n = 90) were randomly distributed into 10 groups (n = 9/group, Table 1) and immunized subcutaneously at the base of tail with the rAPN1 (5 μg/mouse for prime and 2.5 μg/mouse at boosts) alone (non-adjuvanted vaccine group; group 1) or formulated in CpG (10 μg/mouse, vaccine grade type, InvivoGen, San Diego, CA, USA; group 2), MPL (10 μg/mouse, vaccine grade type, InvivoGen, San Diego, CA, USA; group 3), and QS21 (10 μg/mouse, vaccine grade type, InvivoGen, San Diego, CA, USA; group 4), and in a mixture containing CpG (5 μg/mouse), MPL (5 μg/ mouse) and QS21 adjuvants (CMQ, group 5), three times at 14-day intervals. Control mouse groups received 1×PBS alone (group 6) or in CpG ODN (10 μg/mouse; Group 7), MPL (10 μg/mouse; group 8), and QS21 (10 μg/mouse; group 9) adjuvants alone, or as a mixture (CMQ, 5 μg/mouse of each; group 10). For the evaluation of anti-APN1 antibody responses, the sera were obtained from the immunized mice prior to the first injection [as pre-immune sera/normal mouse sera (NMS)] and also on days 10, 24, 38, and 180 after the primary immunization from the end of the mice’s tail (Table 1). The collected sera samples were kept at -20°C for further investigations.

**Table 1:** Mouse immunization strategies.

Group no.	Group	Ag (μg/mouse)			Adjuvants (μg/mouse)		
		Prime*	Boost1**	Boost 2***	CpG	MPL	QS21
**1 (n = 9)**	rAPN1	5	2.5	2.5	-	-	-
**2 (n = 9)**	rAPN1+ CpG	5	2.5	2.5	10	-	-
**3 (n = 9)**	rAPN1 +MPL	5	2.5	2.5	-	10	-
**4 (n = 9)**	rAPN1+QS21	5	2.5	2.5	-	-	10
**5 (n = 9)**	rAPN1 +CpG, MPL, QS21	5	2.5	2.5	5	5	5
**6 (n = 9)**	1× PBS	-	-	-			
**7 (n = 9)**	CpG	-	-	-	10	-	-
**8 (n = 9)**	MPL	-	-	-	-	10	-
**9 (n = 9)**	QS21	-	-	-	-	-	10
**10 (n = 9)**	CpG, MPL, QS21	-	-	-	5	5	5

*Day 0

**Day 14

***Day 28.

Groups 1–5: Vaccinated groups.

Groups 6–10: Negative controls.

Female BALB/c mice (n = 90) were randomly distributed into ten groups and immunized subcutaneously at the base of the tail with 100 μl antigen (5 μg in prime and 2.5 μg at boosts) alone, as a non-adjuvanted vaccine group, or in combination with distinct adjuvants, CpG ODN (10 μg/mouse), MPL (10 μg/mouse) and QS21 (10 μg/mouse), alone and as a mixture (CMQ [CpG/MPL/QS21]) (5 μg/mouse of each), as adjuvanted vaccine groups. Control mouse groups (groups 6–10) received 1×PBS alone or in combination with CpG ODN (10 μg/mouse), MPL (10 μg/mouse), and QS21 (10 μg/mouse) alone and as a mixture (CMQ) (5 μg/mouse of each). Sera samples were collected from the tail vein on days 10, 24, 38, and 180 after the first immunization.

### ELISA-based measurement of antibody responses to rAPN1

Anti-APN1 antibodies in the collected sera from pre-immunization, days 10 (10 days after the primary immunization), 24 (10 days after the boost 1), 38 (10 days after the boost 2), and 180 were evaluated using ELISA, as described previously [29,30]. To optimize the antigen concentrations and the dilution of the primary and secondary antibodies, a checkerboard cross-titration assay was performed using the following factors. The antigen concentration ranged from 10 to 200 ng and serum dilutions from 1: 200 to 1: 3200. The optimized concentration of APN1 (8 ng/well) was added into MaxiSorp flat-bottom 96-well ELISA plates (Jet Biofil, Guangzhou, China) and incubated at 4°C overnight. After blocking, serum samples were added to the desired wells at 1:800 dilutions. After incubation and rinsing wells, a secondary HRP-conjugated goat anti-mouse IgG antibody was added at 1:25000 dilution. Anti-APN1 IgG was detected using 3,3’,5,5’-Tetramethylbenzidine (TMB) as a substrate. The reaction was stopped with 2 N H_2_SO_4_, and OD_450_nm was measured using an ELISA microplate reader (BioTek, Winooski, VT, USA). To estimate the cut-off values, the mean OD_450_nm of the 20 NMS plus three standard deviations (SD) was calculated.

Furthermore, the anti-APN1 IgG subclasses (IgG1, IgG2a, IgG2b and IgG3) were evaluated by an ELISA as described above; however, after mouse sera incubation, 1: 1000 dilution of the secondary goat antibodies specific to mouse IgG2a and IgG2b, and 1:2000 dilution of IgG1, and IgG3 (Sigma-Aldrich Co.) antibodies were used. Subsequently, the plates were incubated with 1: 10,000 dilution of anti-goat IgG HRP (Sigma-Aldrich, USA) at room temperature (RT) for 1 h. Then, the reaction was continued in the aforementioned procedure. To evaluate the persistence of antibodies, the sera including anti-APN1 antibodies were collected six months after immunization. To determine the persistence and the level of antibodies on day 180, ELISA results were compared with outcomes on day 38 (10 days after the second boost).

### Anti-APN1 antibody avidity and titration

Anti-APN1 IgG avidity and its subclasses (IgG2a and IgG2b) were assessed as described previously [31] with some modifications. ELISA was performed as described above by the pooled mouse serum samples (1:800 dilution) obtained on day 38 (n = 9) after the first immunization using optimal concentration of antigen (rAPN1: 8 ng/well) in two separate plates. All steps were performed similarly to the explained protocol, and after serum incubation, one plate was washed three times with PBS-T and the other plate with wash buffer containing PBS-T and 5 M urea. The avidity index (AI) was expressed by the proportion of urea-treated to non-treated samples multiplied by 100. AI values <30%, 30% -50%, and >50% determine the low-, intermediate-, and high-avidity antibodies, respectively.

Endpoint titers of anti-APN1 IgG, IgG1, IgG2a, and IgG2b antibodies raised in the mice on day 38 after the first immunization were assessed by antibody titration ELISA with pooled sera from each mouse group in serial dilutions (1: 800–1: 1,368,400). The titration endpoints of anti-APN1 total IgG, IgG1, IgG2a, and IgG2b antibodies were considered the last dilution of serum with an OD_450_ value above the cut-off (Mean OD_450_ value + 3SD of NMS).

### Cytokine assay

Mouse splenocytes for each group (n = 4) were isolated and cultured on days 38 and 180 for cytokine analysis. Mice were anesthetized using anesthetic drugs (ketamine and xylazine) in the amount of 2 times the anesthetic dose (anesthetic dose of 80 mg/kg ketamine and 10 mg/kg xylazine) through intraperitoneal injection. Then, mice were euthanized by translocating the cervical vertebrae. After euthanization under sterile conditions, the spleens were removed, and a single-cell suspension was prepared in RPMI 1640 medium (Gibco, Invitrogen, Scotland, UK), then RBCs were removed by ammonium chloride-potassium lysis buffer (pH 7.2). Subsequent, the cells were re-suspended in the complete culture medium containing RPMI 1640 medium (Gibco, USA), 5% fetal calf serum (FCS; Sigma, USA), 2.3 ×10^−2^ mM 2-mercaptoethanol, penicillin-streptomycin (100 U–100 μg/ml), and 10 mM HEPES (Sigma-Aldrich, USA). Then, 100 μL of cells (3 × 10^6^ cells/mL) were grown in a flat-bottom 96-well tissue culture plate (Orange Scientific, EU, Belgium) in four repeats. Splenocytes were incubated with Concanavalin A (5 μg/ml as a positive control), rAPN1 (20 μg/ml), or medium alone (negative control) in a 5% CO_2_ incubator at 37°C.

The supernatants of splenocytes stimulated with APN1 were obtained after 24h for IL-4, 72 h for IL-10 and TNF, and 120h for IFN-γ. Then, the cytokine profiles were measured and analyzed using ELISA kits (IFN-γ (#DY485), TNF (#DY410), IL-10 (#DY417), and IL-4 (#DY404), R&D system, Minneapolis, USA). The concentration of cytokines was calculated based on the standard curves performed in parallel with the known concentrations of recombinant mouse IL-4, IL-10, TNF, and IFN-γ for each experiment. The mean of concentration ± SD was recorded for each set of samples.

### Standard membrane feeding assay (SMFA)

The standard membrane-feeding assay was performed to determine the potency of induced antibodies for the inhibition of oocyst formation in examined mouse groups. For this purpose, the *P*. *falciparum* gametocytes were prepared using *in vitro* continuous culture. To produce gametocytes, the *P*. *falciparum* NF54 strain (a kind gift from the Pasteur Institute of Paris) was cultured continuously on human erythrocytes (RBCs) (O^+^ blood group; Blood Transfusion Organization, Tehran, Iran) in RPMI 1640 medium (Gibco, USA) supplemented with 5% pooled human AB^+^ serum, 0.2% (wt/vol) AlbuMAX II (Gibco, USA), 25 mM HEPES (Sigma, USA), 50 mg/liter of hypoxanthine (Sigma, USA), 1.96 g/liter of glucose, and 25 mM NaHCO_3_ (pH 7.2). Gametocyte induction was performed according to the protocol established by Fivelman et al. in 2007 [32]. The different stages of gametocytes were classified according to the Carter and Miller guidelines [33], and mature gametocytes were harvested 12 days after spent medium stress initiation with a ratio of 1 male: 2–4 female. To confirm the maturity of the gametocytes, the exflagellation test was performed in which the mature male gametocytes were transformed into male flagellated gametes, and scrutinized under a light microscope. *An*. *stephensi* mosquitoes were kept in the National Insectarium of the Pasteur Institute of Iran, Karaj, and bred at 26°C and 80% humidity with a 12/12 light/dark cycle. To test the inhibition of oocyst formation through the SMFA technique, blood-feeding was performed for the 4-5-day-old female mosquitoes (50 per group) that were starved overnight with gametocytes along with the serum of different vaccinated mouse groups in equal percentages. Mosquitoes were allowed to feed blood for 20 minutes, and fully fed *Anopheles* mosquitoes were selected and kept at a temperature of 26°C and a humidity of 80%. For feeding, mature stage *P*. *falciparum* NF54 gametocytes were mixed with washed human O+ blood group at 50% hematocrit in human AB+ serum. An equal volume of pooled mouse sera from each test group (collected on day 38, from 9 mice per group) or control group was added to the mixture. Pooled pre-immune mice sera (n = 30) were utilized in a control group as normal mouse sera (NMS). Blood-fed mosquitoes were nourished continuously with 10% sugar on water-soaked cotton, and on days 8–11, mid-gut infection of the mosquitoes was assessed to detect oocysts after dissection and staining using 0.2% mercurochrome. The stained oocysts were counted under the light microscope, transmission-reducing activity (TRA) and transmission-blocking activity (TBA) were calculated in comparison to the control groups as follows:

TRA = 100(1-Mean number of oocysts in the test group/mean number of oocysts in the control group)

TBA = 100(1-proportion of mosquitos with any oocysts in the test group/ proportion of mosquitos with any oocyst in the control group)

### Statistical analyses

The normality of the ELISA data for each group was confirmed using the Shapiro-Wilk test. Given the normal distribution of the data, differences in antibody levels and cytokine responses among the vaccinated mouse groups were evaluated using one-way ANOVA. To account for multiple comparisons following ANOVA, the Bonferroni post hoc test was employed for pairwise group comparisons. Furthermore, a paired sample t-test was applied to compare antibody levels within each group on days 10, 24, and 38, with p-values adjusted for multiple comparisons using the Bonferroni correction. Regarding the cytokine assays, for comparison of the cytokine levels on days 38, and 180, we employed independent samples t-test due to the distinct mouse cohorts used at each time point, resulting in independent data sets. All statistical analyses were conducted with a significance level set at P <0.05.

For analysis involving SMFA data, which did not follow a normal distribution, a non-parametric Kruskal-Wallis H test was employed to evaluate differences in oocyst production intensity across six independent groups. Following the Kruskal-Wallis H test, multiple comparisons with Bonferroni-Dunn’s correction test were performed. In addition, Fisher’s exact test was utilized to examine differences in the frequency of mosquito infection among groups, and the obtained P values were adjusted with Bonferroni correction analysis. A confidence level of P < 0.05 was considered statistically significant for all analyses.

## Results

### Expression and purification of APN1 protein

**Fig 1:**
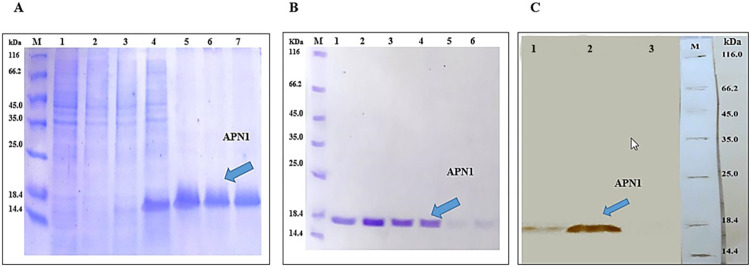
SDS-PAGE and Western blot analysis of APN1. (**A**) SDS-PAGE analysis of purified APN1. Lane M: Molecular weight protein marker (Fermentas, 116–14.4 kDa), lanes 1,2: *E*. *coli* BL21(DE3)-pET23a 3,4: *E*. *coli* BL21(DE3)-pET23a-APN1; lanes 1,3: Before induction; lanes 2,4: 16h after induction with IPTG. Lanes 5–7: Purified APN1 (10 μl/well was loaded). (**B**) SDS-PAGE analysis of desalted purified APN1. Lane M: Molecular weight protein marker (Fermentas, 116–14.4 kDa), Lanes 1–6: Desalted purified APN1. 20 μl of each sample from the desalting process was loaded in each well. (**C**) Western blot analysis of APN1 protein with anti-His tag mAb. Lanes 1 and 2: Before induction and 16h after induction of *E*. *coli* BL21(DE3)- pET23a-APN1, Lane 3: 16h after induction of *E*. *coli* BL21(DE3)-pET23a as negative control and Lane M: Molecular weight protein marker (Fermentas, 116–14.4 kDa) stained with Ponceau S and marked with a pencil (10 μl/well was loaded).

rAPN1 expression was accomplished and purified in *E*. *coli* BL21(DE3) harboring pET23a-APN1 as confirmed using SDS-PAGE analysis, showing an 18 kDa band without any non-specific bands (Fig 1A). To prepare the antigen for mice immunization, purified rAPN1 was successfully desalted. The integrity of the sample’s following purification was confirmed by observing an 18 kDa band on SDS-PAGE. (Fig 1B). For further analysis, western blotting using a His-tag-specific antibody confirmed the presence of rAPN1 in 16 hours post-induction (Fig 1C).

### ELISA-based measurement of antibody responses to rAPN1

**Fig 2:**
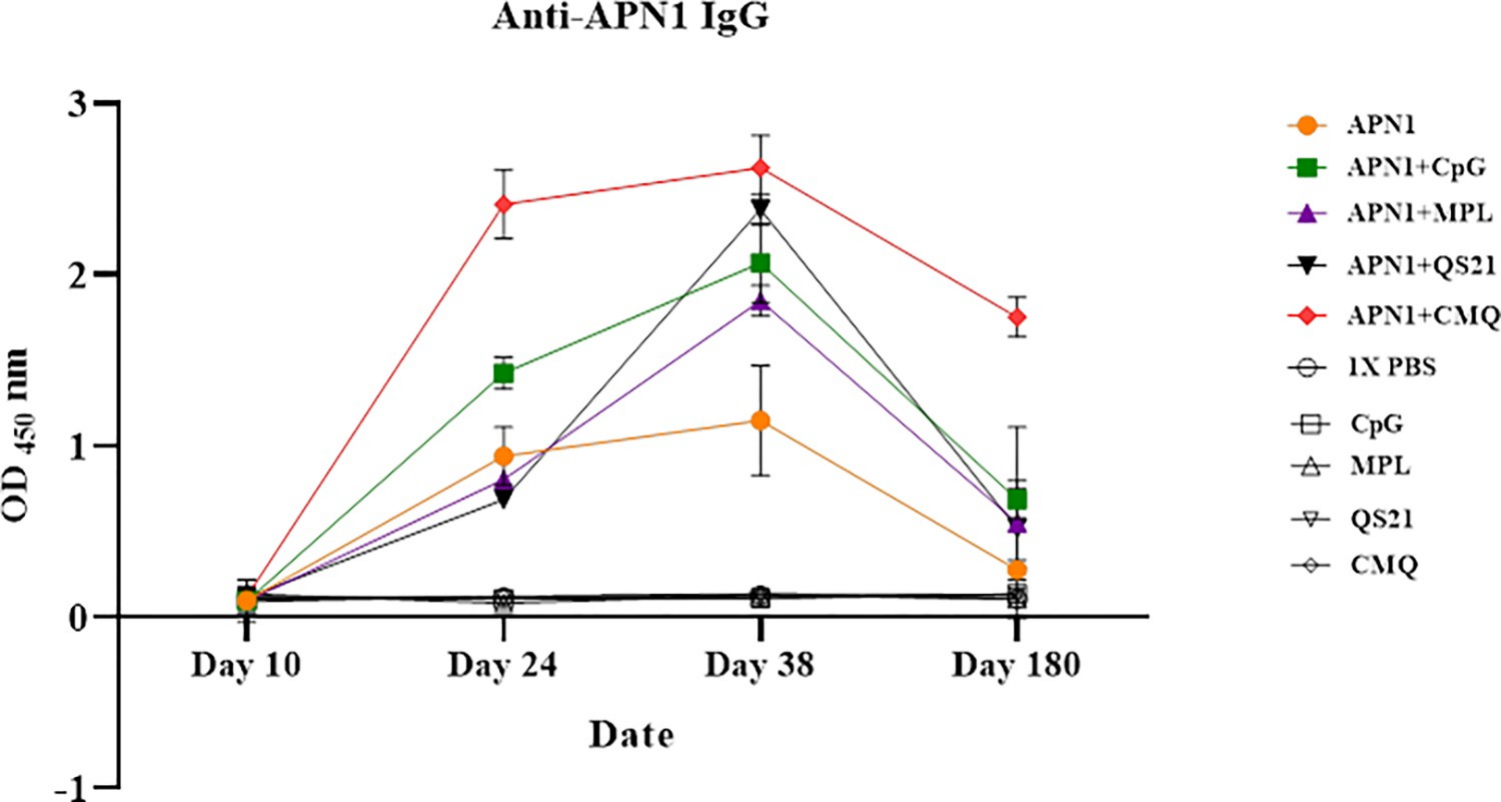
Evaluation of the level of anti-APN1 IgG antibody among vaccinated mouse groups at different time points by an ELISA. Mouse groups were immunized subcutaneously with recombinant APN1 alone or formulated with different adjuvants (CpG, MPL, QS21, and CMQ [CpG/MPL/QS21]). Control groups received 1×PBS alone or with adjuvants. Anti-APN1 IgG antibody levels were compared at different time points on days 10 (n = 9), 24 (n = 9), 38 (n = 9), and 180 (n = 4) after the first immunization in each mouse group. There was a significant difference in total IgG antibody levels between different immunization time points (days 10, 24, and 38) in each vaccine group (Adjusted P <0.05, paired-sample t-test). Comparing the anti-APN1 IgG antibodies in vaccinated mouse groups on day 38 revealed a significant difference (P < 0.0001, one-way ANOVA test), and the highest level of anti-APN1 IgG antibodies was observed in mice receiving APN1/CMQ.

The optimization experiment determined that 5 μg of antigen per mouse is the optimum concentration for immunization (S1 File). In the main test, anti-APN1 IgG antibody responses were measured on days 10, 24, 38, and 180 among vaccinated mouse sera using ELISA. Results showed a significant increase in the level of anti-APN1 IgG antibodies on days 24 and 38 compared to day 10 after primary immunization in all vaccinated groups (groups 1 to 5), respectively (adjusted P < 0.05 by a paired-sample t-test) (Fig 2). No detectable anti-APN1 IgG antibodies were found in the control groups immunized with only adjuvant(s) and 1× PBS. The highest significant level of anti-APN1 IgG was observed in group 5, rAPN1 formulated with three CPG, MPL, and QS21 adjuvants (mean OD_450_nm: 2.622). A significant lower level of IgG was observed in group 1 of mice immunized with rAPN1 alone (mean OD_450_nm: 1.147) in compare to groups 4 (P = 0.014) and 5 (P <0.0001, Bonferroni *post hoc* test, Fig 2). Among the adjuvanted vaccine groups, mice receiving rAPN1/MCQ induced a significant higher level of anti-APN1 IgG antibodies relative to mouse group 3 received rAPN1/MPL (P = 0.040, Bonferroni post hoc test, Fig 2). For determination of the antibody persistence besides the magnitude and durability, we also compared the level of anti-APN1 IgG antibodies on days 38 (after the second boost) and 180 (after six months) of primary immunization (Fig 2). The level of IgG antibody against rAPN1 decreased on day 180, relative to day 38 of the first immunization in all groups 1–5 (Fig 2). On day 180, the highest and lowest levels of anti-APN1 IgG antibodies were observed in vaccine groups 5 (receiving rAPN1/CMQ, Mean OD_450_nm = 1.749) and 1 (receiving rAPN1, Mean OD_450_nm = 0.274), respectively (Fig 2).

**Fig 3:**
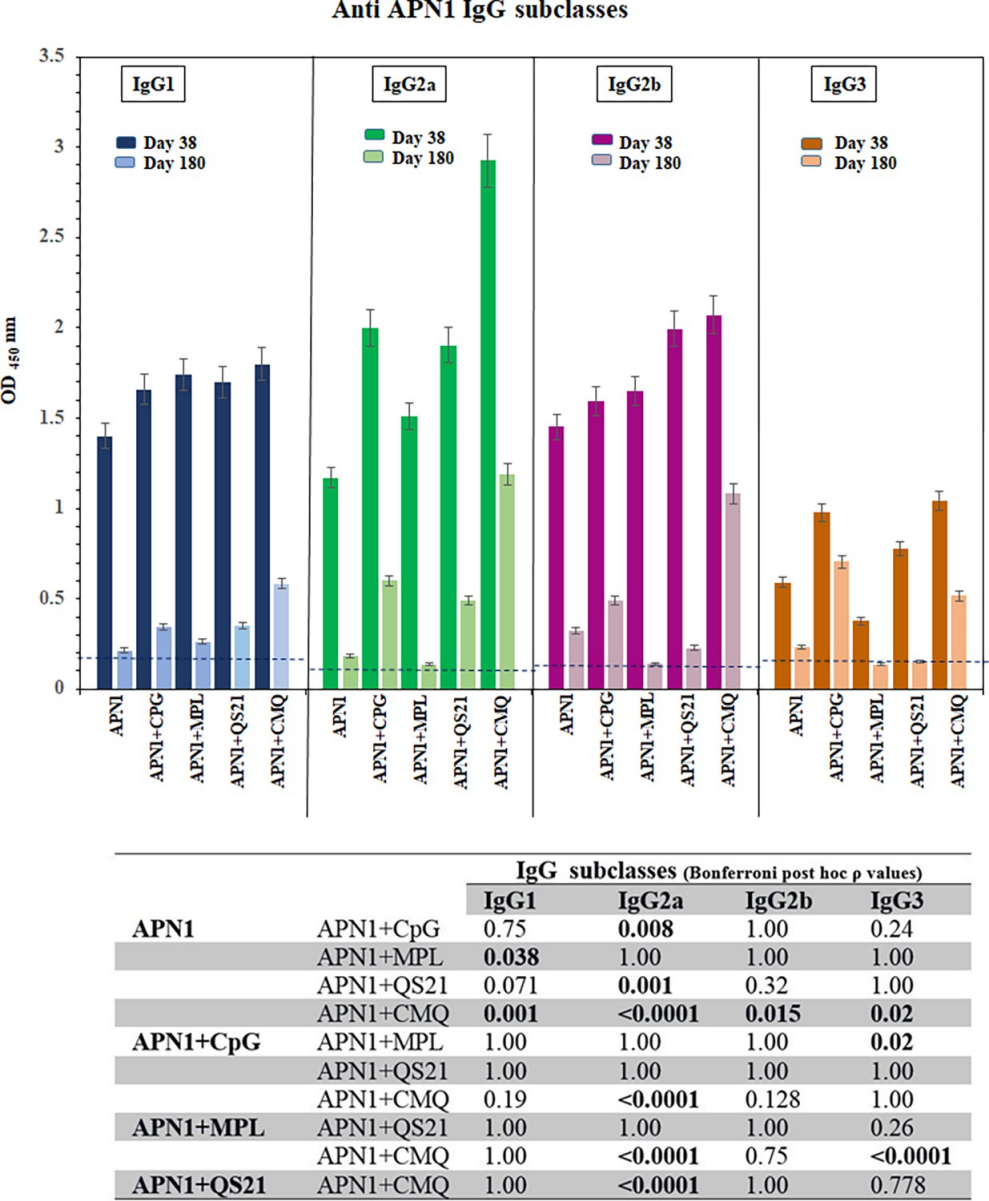
Analysis of the profile and persistence of anti-APN1 IgG subclasses. The profile of anti-APN1 IgG subclasses among vaccinated mouse groups. Pre-immune sera were used as negative controls to determine the ELISA cut-offs. The bars and error bars show the mean OD_450_ values and standard deviations (SD) for 9 individual mice (day 38) and 4 individual mice (day 180) in each group, respectively. The significant difference in the level of anti-APN1 IgG subclasses was observed in vaccine groups (P < 0.05, one-way ANOVA). The table shows multiple comparisons of means for anti-APN-1 subclasses among the non-adjuvanted (G1) and adjuvanted (G 2–5) vaccine groups on days 38 after the first immunization, which was performed using the Bonferroni *post hoc* test.

The profile of anti-APN1 IgG subclasses was analyzed in mouse sera collected from immunized mouse groups on days 38 and 180 following primary immunization. The highest level of anti-APN1 IgG1 (mean OD_450_nm: 1.8), IgG2a (mean OD_450_nm: 2.926), and IgG2b (mean OD_450_nm: 2.07) antibodies were induced in group 5 receiving rAPN1/CMQ. Besides, the mice receiving rAPN1 alone had a lower level of IgG1 (P = 0.001), IgG2a (P < 0.0001), and IgG2b (P = 0.015) antibodies relative to mouse group 5 receiving rAPN1/CMQ (Bonferroni *post hoc* test, Fig 3). In addition, Multiple comparisons of anti-APN1 IgG2a antibodies showed that mouse group 5 receiving rAPN1/CMQ induced a higher level of IgG2a antibodies compared to mice receiving rAPN1 alone or with single adjuvants CpG, MPL, or QS21 (groups 1, 2, 3, and 4, respectively; P < 0.0001, Bonferroni *post hoc* test, Fig 3). Regarding anti-APN1 IgG1 and IgG2b antibodies among adjuvanted vaccine groups, no significant difference was observed in mice immunized with various formulations (P > 0.05, Bonferroni *post hoc* test, Fig 3). Concerning IgG3, a significant difference was found in the level of anti-APN1 IgG3 between vaccine groups 2–5 on day 38 of the first immunization (P < 0.014, one-way ANOVA, Fig 3). To determine the persistence of induced antibodies, the level of anti-APN1 IgG subclasses was measured on day 180. The results illustrated a substantial decrease in the level of subclasses in all vaccinated mouse groups. However, the lowest reduction for IgG1, IgG2a, and IgG2b was observed in group 5 receiving APN1/CMQ (Fig 3).

### High-titer and high-avidity antibodies induced in adjuvanted vaccine groups

**Fig 4:**
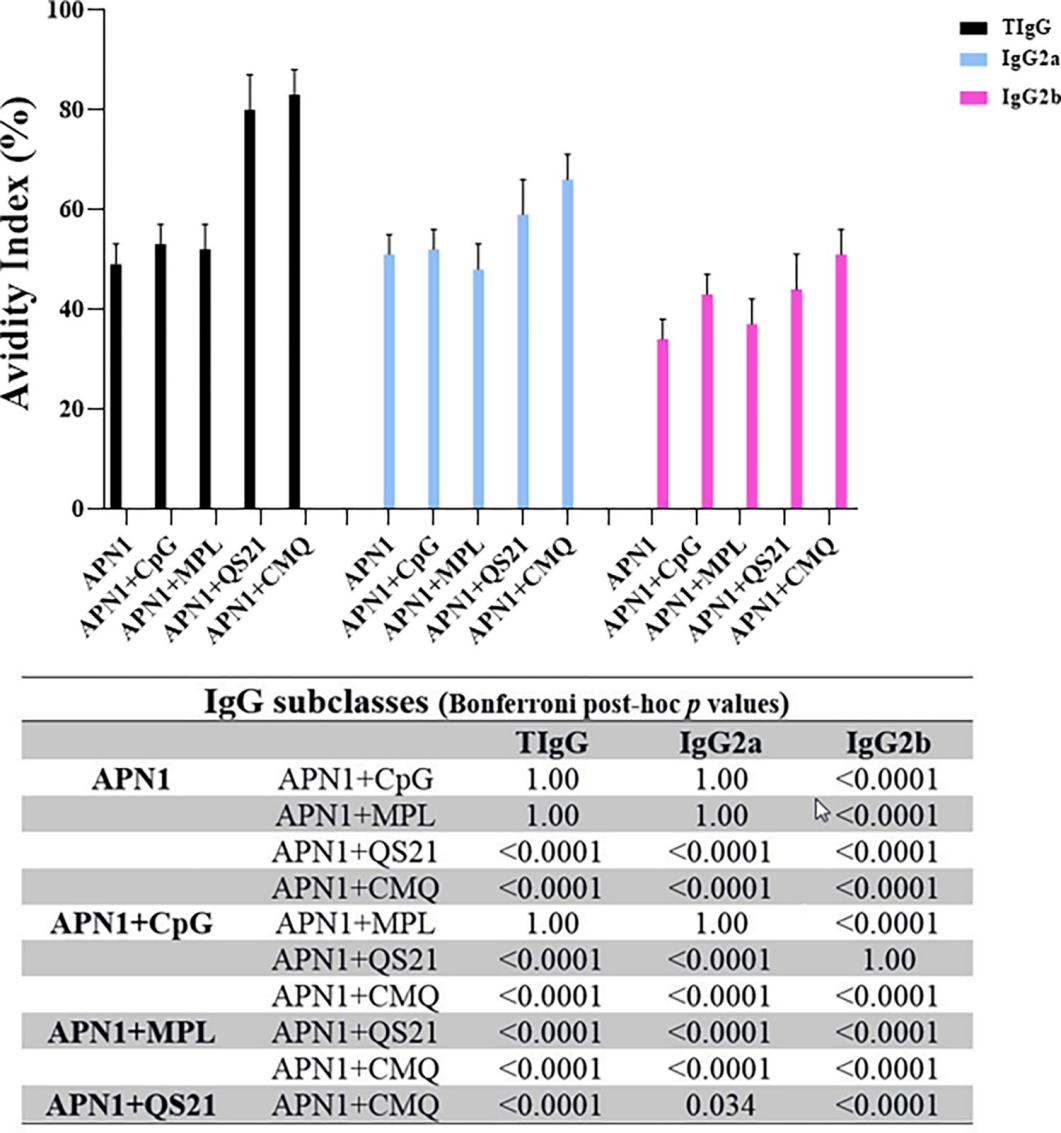
Analysis of the avidity of anti-APN1 IgG, IgG2a, and IgG2b antibodies. The avidity index (AI) was calculated as the portion of the OD value of urea-treated serum samples to that of untreated samples multiplied by 100. AI values <30%, 30 to 50%, and >50% correspond to low, intermediate, and high avidity antibodies, respectively. The highest AI of anti-APN1 IgG, IgG2a, and IgG2b antibodies was observed in vaccine group 5 (APN-1/CMQ [CpG/MPL/QS21]) on day 38 after the first immunization. The table shows multiple comparisons of means for the avidity indices of anti-APN-1 subclasses among the non-adjuvanted (G1) and adjuvanted (G 2–5) vaccine groups on days 38 after the first immunization, which was performed using the Bonferroni *post hoc* test.

In this study, high-avidity anti-APN1 IgG antibodies were induced in the adjuvanted vaccine groups (G2-5) receiving rAPN1 antigen formulated in different adjuvants on day 38 of the primary immunization. However, mice immunization with rAPN1 antigen without any adjuvant resulted in intermediate-avidity for IgG antibodies on day 38 of the first immunization (Fig 4). Multiple comparisons of AI for IgG among the adjuvanted vaccine groups 2–5 revealed that high-avidity IgG antibody in the mouse group 5 (rAPN1/CMQ) was significantly different from the mouse groups 2–4 (rAPN1/MPL, rAPN1/CpG, and rAPN1/QS21) on day 38 of the first immunization (P < 0.0001, Bonferroni post hoc test).

Regarding IgG2a, all vaccine groups except G3 (receiving antigen plus MPL) elicited high avidity IgG2a antibodies. In addition, mouse group 5 receiving rAPN1 plus CMQ showed high avidity IgG2b antibodies; however, mice immunized with rAPN1 alone or formulated with single adjuvants were categorized in intermediate-avidity antibodies. The highest avidity index (AI) for IgG, IgG2a, and IgG2b subclass antibodies was observed in the mouse group 5 receiving rAPN1 in CMQ mixture adjuvants (Fig 4).

**Fig 5:**
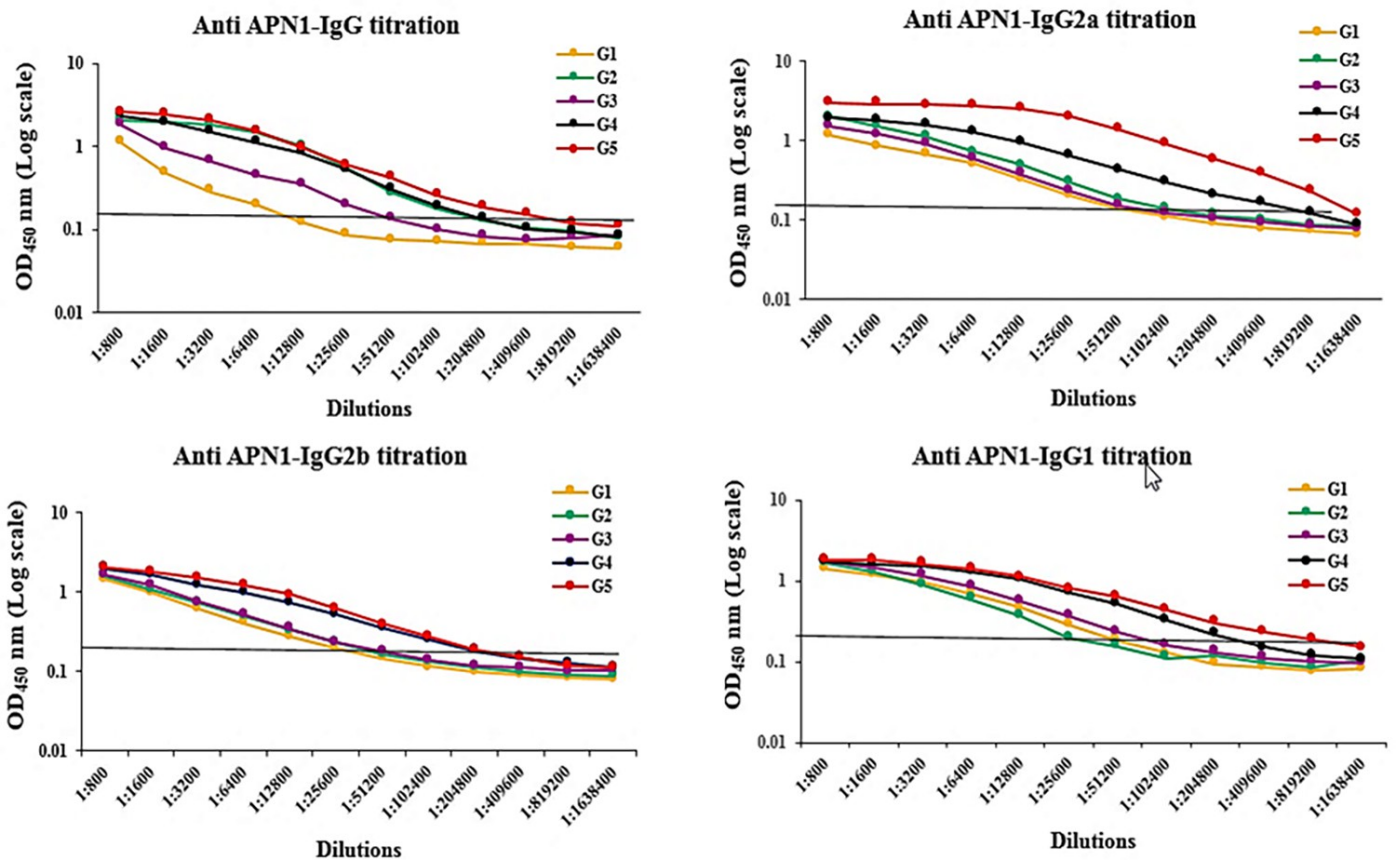
Evaluation of end-point titers of anti-APN1 IgG, IgG1, IgG2a, and IgG2b antibodies among vaccine groups. For titration, 1:800 to 1:1,638,400 dilutions of mouse sera from different vaccine groups (groups 1 to 5) were analyzed using an ELISA. The titer was calculated as the last dilution of test sera in which OD_450_ nm values were above that cut-off. Among all the vaccine groups (groups 1 to 5), the highest endpoint titers of anti-APN1 total IgG, IgG1, IgG2a, and IgG2b were detected in mouse group 5 receiving APN1/CMQ (CpG/MPL/QS21) on day 38 after the first immunization. The cut-off values were calculated as the mean OD_450_nm of the 20 NMS plus three standard deviations (SD). The cut-off values are as follows: IgG: 0.17, IgG1: 0.132, IgG2a: 0.138, and IgG2b: 0.16. The cut-offs are shown with gray lines.

Among all the vaccine groups 1–5, the highest end-point titer of anti-APN1 total IgG was detected in the mouse group 5 (1: 204,800) receiving APN1/CMQ on day 38 of the first immunization (Fig 5). However, the lowest end-point titer of IgG (1:6400) was detected in the mouse vaccine group 1 (receiving APN1 alone). Analysis of anti-APN1 IgG1 titer in vaccine groups showed the highest endpoint titer (819,200) in group 5 receiving APN1 with CMQ adjuvants, while the lowest endpoint titer (51,200) was detected in mouse groups 1 and 2 receiving APN1 alone or in combination with CpG adjuvant, respectively (Fig 5).

In the case of IgG2a, the mouse groups that received APN1/CMQ induced the highest end-point titer of 819,200, followed by G4 (409,600), G2 (102,400), G3 (51,200), and G1 (25,600). Regarding IgG2b, the highest end-point titer was detected in groups 5 (APN1/CMQ) and 4 (APN1/QS21) with an end-point titer of 204,800. However, immunization of mice with APN1 alone (group 1) induced the lowest end-point titer (25,600, Fig 5).

### Cellular immune responses

**Fig 6:**
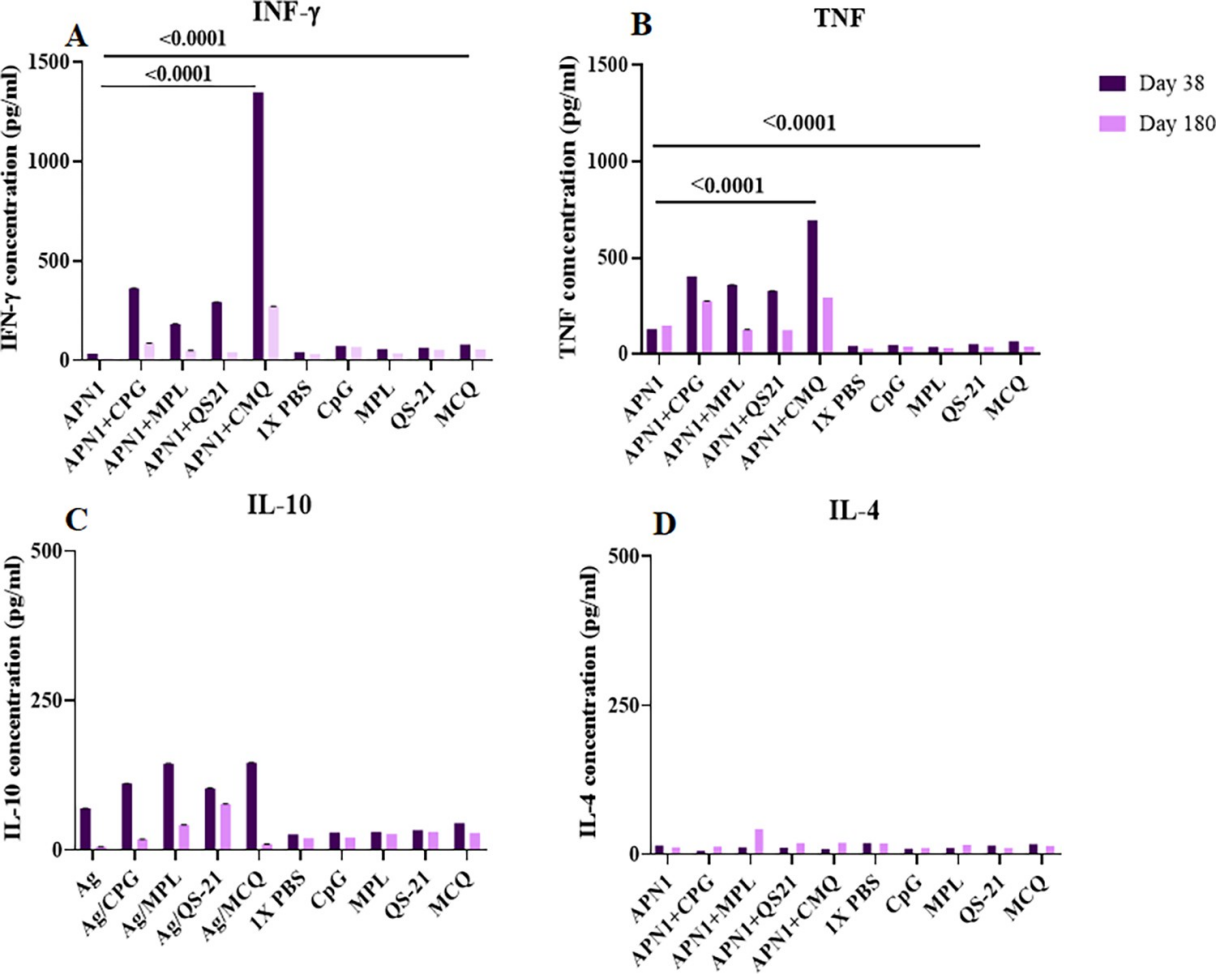
Assessment of IFN-γ (A), TNF (B), IL-10 (C), and IL-4 (D) production in vaccine groups (G 1–5) and control groups (G 6–10) on days 38 and 180 after primary immunization. For immunized mice receiving APN1 with different adjuvants alone or in combination, the mean IFN- γ responses in the presence of ConA (as the positive control) and no antigen (as the negative control) were 1426 and 6 pg/ml, respectively. In addition, the mean TNF response in the presence of ConA (as the positive control) and no antigen (as the negative control) were 161 and 30 pg/ml, respectively. Besides, IL-10 responses in the presence of ConA (as the positive control) and no antigen (as the negative control) were 374 and 31 pg/ml, respectively. Moreover, IL-4 responses in the presence of ConA (as the positive control) and no antigen (as the negative control) were 48.497 and 28.37 pg/ml. On day 180 after primary immunization, the levels of all cytokines were significantly reduced in all the vaccine mouse groups (*P* < 0.05, Independent-samples t-test).

The analysis of cytokine profiles revealed eliciting significant levels of IFN-γ in all the adjuvanted vaccine groups (G2-5) as compared to the mouse control groups on days 38 and 180 of the primary immunization (P < 0.0001, one-way ANOVA, Fig 6). Among vaccine groups 1–5, the highest and the lowest levels of IFN-γ production were detected in immunized mouse group 5 (APN1/CMQ = 1347.7 and 271 pg/ml) and non-adjuvanted vaccine group 1 (APN1 = 33 and 11 pg/ml) on days 38 and 180 of the first immunization, respectively (P < 0.0001, one-way ANOVA, Fig 6A). Additionally, multiple comparisons among the adjuvanted vaccine groups 2–5 showed that the level of IFN-γ in the vaccine group 5 was significantly higher than vaccine groups 2–4 on days 38 and 180 of the primary immunization (P < 0.0001, Bonferroni post hoc test, Fig 6A). On day 180 after the primary immunization, the level of IFN-γ significantly decreased in all the vaccine groups 1–5 (P < 0.05, Independent-samples *t*-test, Fig 6A). However, a significant level of IFN-γ was still observed in the mouse group 5 receiving rAPN1 formulated in CMQ mixture adjuvants (271 pg/ml, Fig 6A).

The analysis of the TNF production showed a significant difference between the vaccine groups 1–5 and the control groups (P < 0.0001, one-way ANOVA, Fig 6B). The highest and the lowest levels of TNF were produced by vaccine group 5 (693.6 and 292.9 pg/ml) and group 1 (129.9 and 148.2 pg/ml) on days 38 and 180 of the first immunization, respectively (P < 0.05, one-way ANOVA, Fig 6B). The level of TNF in the mouse group 5 (rAPN1/CMQ) was significantly higher than the mouse groups 2–4 (rAPN1/MPL, rAPN1/CpG, and rAPN1/QS21, respectively) on days 38 of the primary immunization (P < 0.05, Bonferroni HSD post hoc test, Fig 6B). Comparing the reduction of TNF production on day 180 in all the mouse vaccine groups 1–5 revealed that the highest level of TNF was still produced by the splenocytes of the mouse groups 2 and 5 receiving APN1 with CpG or CMQ adjuvants, respectively (Fig 6B).

Analysis of IL-10 (as a regulatory cytokine) showed a low level of IL-10 production in all the mouse vaccine groups (1–5) on days 38 and 180 of the first immunization. There was a significant difference in the level of IL-10 between the vaccine groups 1–5 (mean IL-10 concentration: 69–146 pg/ml) and the control groups 6–10 (mean concentration: 26.4–45 pg/ml) on day 38 and 180 of the primary immunization (P < 0.05, one-way ANOVA, Fig 6C). Regarding the levels of IL-4 secretion, the low levels of IL-4 with no significant difference were detected in different immunized groups (1–5) and the control mouse groups (6–10) (P > 0.05, one-way ANOVA, Fig 6D).

### Transmission-reducing activity (TRA) of anti-APN1 antibodies induced in different vaccine groups

**Fig 7:**
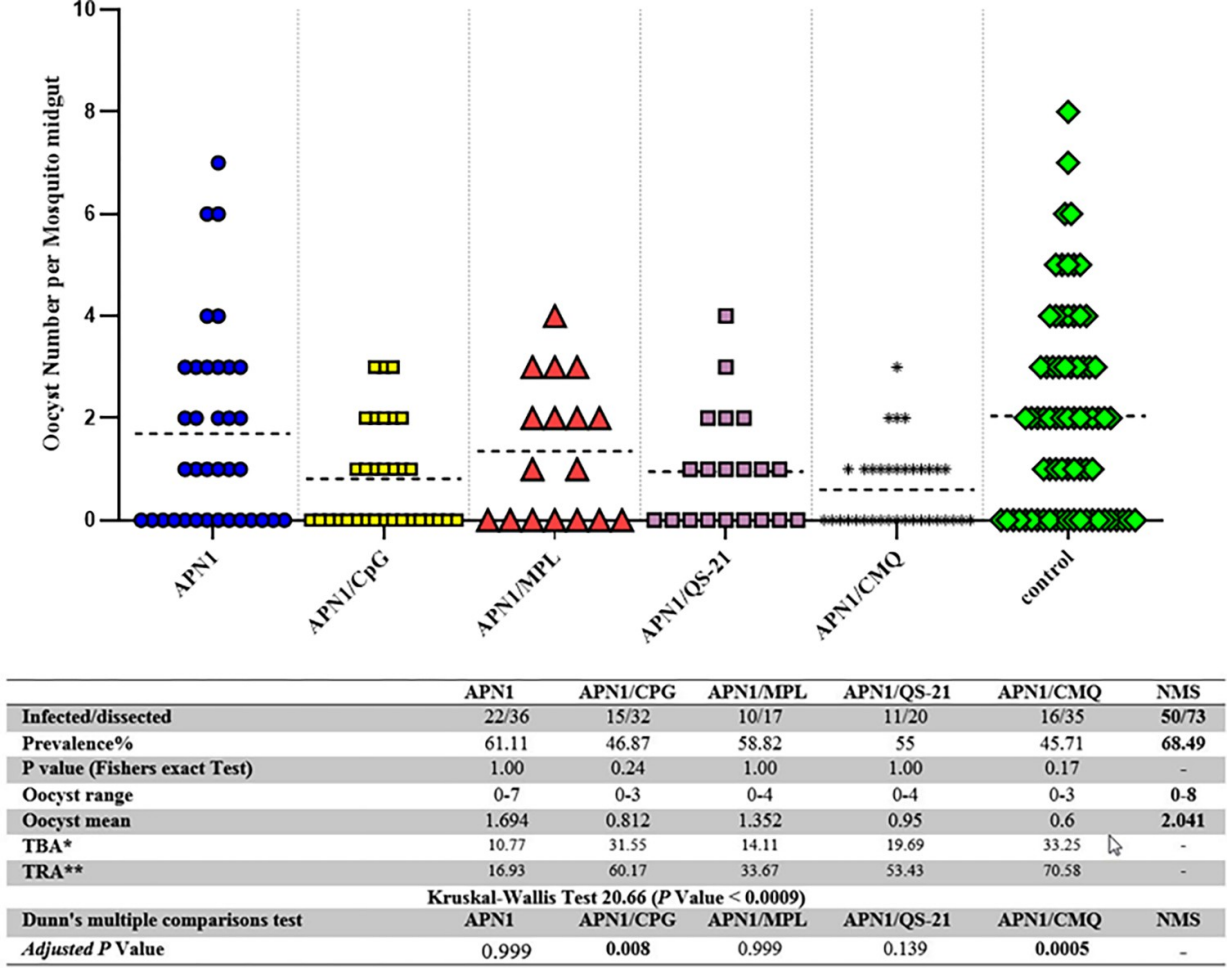
Inhibition of *P*. *falciparum* NF54 parasite development by anti-rAPN1 polyclonal antibodies in *An*. *stephensi* mosquitoes. Pooled mouse sera (n = 9) from different vaccine groups (groups 1 to 5) collected on day 38 after the first immunization were admixed with mature *P*. *falciparum* NF54 cultured gametocytes and fed to *An*. *stephensi* mosquitoes (n = 50/cup) in standard membrane feeding assay (SMFA). Pooled normal mouse serum (NMS) (n = 30 randomly selected from 160 female BALB/c mice before immunization) was used as the negative control. On days 8–10 after feeding, the mosquitoes’ midguts were dissected and oocysts counted, which revealed the successful development of *P*. *falciparum* oocysts in *An*. *stephensi* mosquitoes were recorded. Two separate membrane feeds were done using serum from each vaccine group (groups 1 to 5), and oocyst counts were pooled for statistical analysis. The dots represent the numbers of oocysts in individual mosquitoes, and the vertical lines indicate the arithmetic means of oocyst counts, respectively. The prevalence of infected mosquitoes in the vaccine groups (groups 1 to 5) and the control group (NMS), range of oocyst numbers, mean number of oocysts, and inhibition percent relative to the NMS control group are indicated in the table. The statistical differences in infection prevalence between each test group and control group were analyzed using Fisher’s exact test and the adjusted P values were presented. The Kruskal-Wallis H test yielded a value of 20.66, with an asymptotic significance of <0.0009, Followed by multiple comparisons with the Bonferroni-Dunn’s correction test. P < 0.05 was considered statistically significant and shown in bold. *: TRA = 100 (1-Mean number of oocyst in the test group/mean number of oocyst in the control group).

The effect of Anti-APN1 antibodies, elicited from immunized mouse groups (G1-5), on inhibition of oocyst formation of *P*. *falciparum* NF54 parasite in *An*. *stephensi* mosquitoes were evaluated using the SMFA. The results revealed that induced antibodies in different mouse groups had diverse levels of reduction in oocyst intensity of *P*. *falciparum* infection in *An*. *stephensi* (Fig 7). Similar oocyst intensity and infection prevalence were observed in mosquitoes fed by Normal mouse serum (NMS) or control groups 6–10 (S2 Fig), which show adjuvanted control groups had no inhibitory effect on TRA or TBA.

The mouse sera collected from adjuvanted vaccine groups had various inhibitory efficiencies in oocyst formation (TRA: 33–70.58%). However, the anti-APN1 antibodies from vaccine groups 5 (APN1/MCQ, P = 0.0005) and 2 (APN1/CpG, P = 0.008) significantly inhibited oocyst formation in *An*. *stephensi* relative to the NMS control group (multiple comparisons with Bonferroni-Dunn’s correction test, Fig 7). The mean oocyst intensity significantly reduced from 2.041 in the NMS control group to 0.6 in vaccine group 5 (P = 0.0005, multiple comparisons with Bonferroni-Dunn’s correction test). In addition, the polyclonal anti-APN1 antibodies from the mouse group 5 (APN1/CMQ) showed the highest oocyst inhibition (70.58%) (Fig 7). Proportion analysis of infected mosquitoes in different groups revealed that among adjuvanted vaccine groups, the highest and lowest infection prevalence was detected in mouse group 1 (61% infection) and 5 (45% infection), respectively. Howvere, it was not statistically significant (adjusted P > 0.05, Fisher’s exact test, Fig 7). The infection rate in mosquitoes fed by NMS was 68%.

## Discussion

Subunit recombinant TBV has been proposed as a promising strategy for malaria elimination and eradication. This study evaluated the immunogenicity and functional activity of rAPN1 with MPL, CpG, and QS21 adjuvants in a murine model. The prokaryote-expressed rAPN1 stimulated immune responses in BALB/c mice, and the use of three adjuvants increased the proportion and avidity of antibodies. These high-affinity and long-lasting antibodies interfered with the oocyst development of *P*. *falciparum* in the midgut of *An*. *stephensi*.

Mice immunized with rAPN1 and single adjuvants showed a significant increase in anti-APN1 IgG levels, particularly in group 4 (rAPN1+QS21), after the second boost. However, the highest total IgG was observed in group 5, which received rAPN1 with a combination of adjuvants (CMQ). Adjuvants improved anti-rAPN1 antibody responses, as indicated by higher levels of IgG2a and IgG2b in group 5. This group also demonstrated the most transmission-reducing activity (~70%), suggesting these antibodies may bind to APN1 antigen on the midgut epithelium and interfere with ookinete bindings to APN1 in *An*. *stephensi* mosquitoes. This is following a previous study to evaluate the immune responses against TBV candidate PfCelTOS formulated with similar adjuvants [4] and suggests that the adjuvant combination can stimulate transmission-blocking antibodies in an APN1-based vaccine, possibly by inhibiting oocyst development via binding to the mid-gut epithelium.

Inhibition of the sexual life cycle is performed through the induction of humoral and cellular immune responses [34]. Upon blood feeding, the host immune system components, enter the mosquito’s stomach and can defend against different parasites for hours, reducing transmission in the mosquito’s mid-gut [35,36]. Effective antibody responses against TBVs include neutralization, opsonization, and lysis due to the complement system [37,38]. Neutralizing anti-APN1 antibodies can prevent ookinete binding to the midgut epithelium cells. The cytokine profile stimulated by the rAPN1 antigen showed that it is in alignment with the humoral immune activity in immunized mouse groups, with the highest levels of TNF and IFN-γ along with anti-APN1 IgG, IgG2a, and IgG2b antibodies in group 5, implying the neutralizing activity of these antibodies which suggests that rAPN1 formulated with CMQ combination adjuvants could be an effective formulation for an APN1-based TBV.

The quality of specific-elicited antibodies has a critical role in the protective immunity achieved by the antigen-based TBV via inhibition of sexual development in the mid-gut of *Anopheles* [39]. This study found that the immunized vaccine group 5 (APN1/CMQ) exhibited high-avidity specific IgG antibodies, confirmed by the highest functional activity indicating possible strong binding of elicited anti-APN1 antibodies to native APN1 in the *An*. *stephensi* midgut. The supreme function of the used adjuvants to activate high avidity responses and facilitate protection has been reported against malaria in small animal models [9,11,13,14,27]. The rAPN1/CMQ vaccinated group demonstrated enhanced responses and increased avidity towards IgG2a and IgG2b antibodies. High-affinity anti-APN1 antibodies in group 5, with the highest TRA, suggest potent neutralizing activity.

Subunit antigen-based vaccines have demonstrated partial or unsuccessful protection as TBV candidates necessitate the utilization of proper, potent, and robust adjuvants. In a previous study, the functional activity of AnAPN1 was assessed through the SMFA in *An*. *gambiae*, a potent vector in African malaria settings [9]. They discovered that recombinant AnAPN1 TBV, including peptides 7 and 9 collaborate in stimulating humoral immune response and potentiate transmission-blocking activity, whereas peptide 1 includes an immune-dominant mouse CD4^+^ T cell epitope that functioned as an “immune decoy” and produces remarkable humoral responses without protective antibody titer [9]. In the current study, the qualitative evaluation of the antibody responses to *An*. *stephensi r*APN1 antigen (including peptides 1, 7, and 9) was performed to determine the inhibitory activity for oocyst development in *An*. *stephensi*. SMFA experiment demonstrated that the elicited antibodies against bacterial-expressed rAPN1 protein in vaccinated groups 2 and 5 had a significant functional activity on the transmission of *P*. *falciparum* (NF54) in *An*. *stephensi* mosquitoes. The highest inhibitory activity of group 5 formulated with different TLR agonists and QS21 adjuvants; implying the role of adjuvants on the quality of induced immune responses. However, the difference between functional activity of elicited antibodies in groups 2 and 5 was not statistically significant. Therefore, in terms of safety and cost, it can be concluded that CpG is a better adjuvant to combine with AsAPN1 antigen to induce inhibitory antibodies.

It should be considered that mice and other small models produce dissimilar immune responses with divergent antibody features as compared to non-human primates and humans [40]. Therefore, the efforts on the development of APN1-based vaccines in clinical trials for the further achievement of human-used malaria vaccines should be under vigorous development [9]. The vaccine mixture in this study was designed to stimulate specific, high-avidity, long-lasting antibodies against APN1 and effective TRA in the mosquito mid-gut. The results validate rAPN1/CpG and rAPN1/CMQ as a potent TBV formulation. Among these formulations, rAPN1/CMQ with the highest inhibitory activity and induction of persistent antibodies is recommended in the first priority. However, further improvements could be made by incorporating other TBV candidates as immunogens or using novel adjuvants or delivery systems.

## Supporting information

S1 Fig Sequence alignment of the expressed AsAPN1 (MF143582, 58–196) with AgAPN1 (MK252101.1) sequence. (DOCX)

S2 Fig Comparative analysis of oocyst development in Normal Mouse Sera (NMS) and control groups 6–10 receiving 1×PBS, CpG, MPL, QS-21, or CMQ adjuvants, respectively. (DOCX)

S3 Fig Original figures of SDS-PAGE and western blotting. (DOCX)

S1 File Immune responses in mice immunized with different concentrations of APN1 antigen. (DOCX)
