# Supplementary material for: Malaria transmission blocking activity of Anopheles stephensi alanyl aminopeptidase N antigen formulated with MPL, CpG, and QS21 adjuvants
Source: PLoS One. 2024 Jul 5;19(7):e0306664. doi: 10.1371/journal.pone.0306664 (PMC11226095; doi:10.1371/journal.pone.0306664)
Supplement: S1 Fig — (DOCX) [file pone.0306664.s001.docx]

**58**

An.stephensi VGIVPAQPVNEAYRLPKTSVPIHYDLHLTTEIHRNERQFSGSVAIQLQVLETTDQLVLHTRGLVISSARISSL

An.gambiae IGVAPAQAVDERYRLPTTSIPIHYDLHLRTEIHRNERTFTGTVGIQLQVVQATDKLVMHNRGLVMSSAKVSSL

:*:.***.*:* ****.**:******** ******** *:*:*.*****:::**:**:*.****:***::***

**196**

An.stephensi PNGVTGAPVLIGDATYSTNTTIEHITFTSPNILQPGFFWLEVSFVGQLATNDDGFYVSSYVADSGE

An.gambiae PNGVTGAPTLIGDVQYSTDTTFEHITFTSPTILQPGTYLLEVAFQGRLATNDDGFYVSSYVADNGE

********.****. ***:**:********.***** : ***:* *:****************.**

Figure S1. Sequence alignment of the expressed AsAPN1 (MF143582, 58-196) with AgAPN1 (MK252101.1) sequence. Stars indicate positions that have fully conserved residues; Boxes are denoted APN1 peptides 1, 4, 5, 7 and 9. The dark blue box denoted peptide 1, red for peptide 4, green: peptide 5, light blue: peptide 7, and yellow: peptide 9.
