# Supplementary material for: Malaria transmission blocking activity of Anopheles stephensi alanyl aminopeptidase N antigen formulated with MPL, CpG, and QS21 adjuvants
Source: PLoS One. 2024 Jul 5;19(7):e0306664. doi: 10.1371/journal.pone.0306664 (PMC11226095; doi:10.1371/journal.pone.0306664)
Supplement: S2 Fig — (DOCX) [file pone.0306664.s002.docx]

**Figure S3.** Comparative Analysis of Oocyst Development in Normal Mouse Sera (NMS) and control groups 6-10 receiving 1×PBS, CpG, MPL, QS-21, or CMQ adjuvants, respectively. The graph illustrates the distribution of oocyst counts (represented by dots) in NMS and control groups, following a Standard Membrane Feeding Assay (SMFA). Mouse sera were collected on day 38 post-initial immunization and combined with mature *P. falciparum* NF54 cultured gametocytes. This mixture was fed to *An. stephensi* mosquitoes. Oocyst counts were analyzed by dissecting the mosquitoes’ midguts between days 8 and 10 post-feeding. Statistical analysis revealed no significant difference in mean oocyst counts between NMS and the control groups (P > 0.05, Multiple comparison with the Bonferroni-Dunn’s correction test).
