# Supplementary material for: Malaria transmission blocking activity of Anopheles stephensi alanyl aminopeptidase N antigen formulated with MPL, CpG, and QS21 adjuvants
Source: PLoS One. 2024 Jul 5;19(7):e0306664. doi: 10.1371/journal.pone.0306664 (PMC11226095; doi:10.1371/journal.pone.0306664)
Supplement: S3 Fig — (DOCX) [file pone.0306664.s003.docx]

**Figure S4. Original figures of SDS-PAGE and western blotting**

**B)**

**1 2 3 4 5 6 7 8 9**

**A)**

**1 2 3 4 5 6 7**


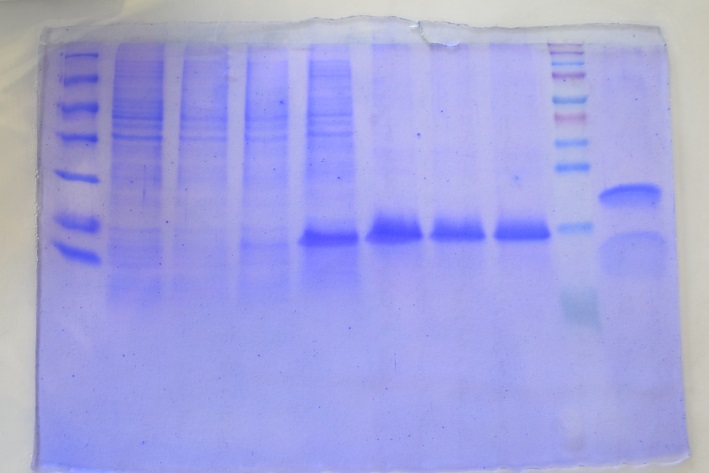

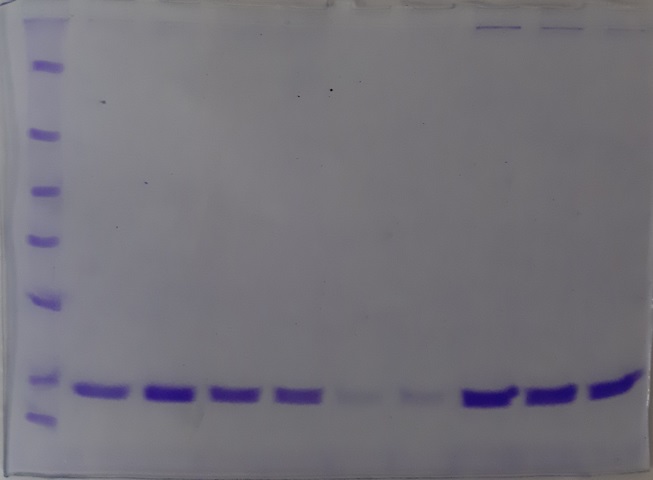


**C)**

**1 2 3 4**


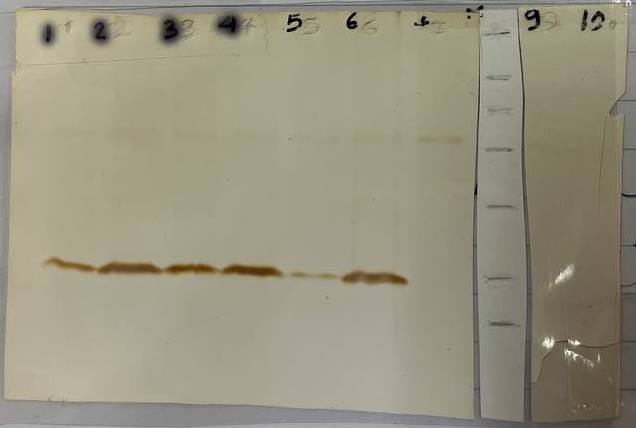


**Figure S4. SDS-PAGE and Western blot analysis of APN1.** (**A**) SDS-PAGE analysis of purified APN1. Lane 1: molecular weight protein marker (Fermentas, 116–14.4 kDa), lanes 2,3: *E. coli* BL21(DE3)-pET23a 4,5: *E. coli* BL21(DE3)-pET23a-APN1; lanes 1,3: Before induction; lanes 2,4: 16h after induction with IPTG. Lanes 5-7: purified APN1. (**B**) SDS-PAGE analysis of desalted purified APN1. Lane 1: molecular weight protein marker (Fermentas, 116–14.4 kDa), Lanes 2–6: desalted purified APN1. (**C**) Western blot analysis of APN1 protein with anti-His tag mAb. Lanes 1 and 2: Before induction and 16h after induction of *E*. *coli* BL21(DE3)- pET23a-APN1, Lane 3: 16h after induction of *E*. *coli* BL21(DE3)-pET23a as negative control and Lane 4: molecular weight protein marker (Fermentas, 116–14.4 kDa) stained with Panso S and marked with a pencil.
