# Supplementary material for: Malaria transmission blocking activity of Anopheles stephensi alanyl aminopeptidase N antigen formulated with MPL, CpG, and QS21 adjuvants
Source: PLoS One. 2024 Jul 5;19(7):e0306664. doi: 10.1371/journal.pone.0306664 (PMC11226095; doi:10.1371/journal.pone.0306664)
Supplement: S1 File — (DOCX) [file pone.0306664.s004.docx]

**Supplementary file S2.**

**Immune responses in mice immunized with different concentrations of APN1 antigen**

Table S1- Mouse immunization strategies in dose optimization for rAPN-1

| Total volume /mouse | **Adjuvants (μg/mouse)** | | | **Ag (μg/mouse)** | | | Groups  n=3 | Group no. |
| --- | --- | --- | --- | --- | --- | --- | --- | --- |
|  | 2^nd^boost (IFA) | 1^st^boost (IFA**) | Prime (CFA*) | **2^nd^ boost** | **1^st^ boost** | **Prime** |  |  |
| 100 µl | 50 µl | 50 µl | 50 µl | 10 µg | 10 µg | 20 µg | APN1(20)+Ad | 1 |
| 100 µl | 50 µl | 50 µl | 50 µl | 5 µg | 5 µg | 10 µg | APN1(10)+Ad | 2 |
| 100 µl | 50 µl | 50 µl | 50 µl | 2.5 µg | 2.5 µg | 5 µg | APN1(5)+Ad | 3 |
| 100 µl | - | - | - | 10 µg | 10 µg | 20 µg | APN1(20) | 4 |
| 100 µl | - | - | - | 5 µg | 5 µg | 10 µg | APN1(10) | 5 |
| 100 µl | - | - | - | 2.5 µg | 2.5 µg | 5 µg | APN1(5) | 6 |
| 100 µl | - | - | - | - | - | - | PBS*** | 7 |
| *CFA:  **IFA:  *** PBS: Phosphate buffer saline | | | | | | | | |

**A) Measurement of anti APN1 Total IgG level in mice vaccinated with different doses of APN1 antigen**

**Figure 1 - IgG antibody response to the recombinant APN1 protein, two weeks after the third injection in the test groups of mice for the optimization test.** Each column shows the mean OD and the error bars show the standard deviation (SD). Group 1: 20 μg of APN1+ CFA/IFA antigen, Group 2: 10 μg of APN1+ CFA/IFA antigen, Group 3: 5 μg of APN1+ CFA/IFA antigen, Group 4: 20 μg of APN1 antigen, Group 5: 10 Micrograms of APN1 antigen, group 6: 5 micrograms of APN1 antigen.

**B) Optimizing the amount of rAPN1 antigen for stimulating the splenocytes by Measurement of the level of cytokines**

The results showed that in the case of IL-4 and IL-10, by changing the amount of antigen, there is no change in the level of these cytokines. In the case of IFN-γ and TNF-α, it was found that the best concentration for stimulating lymphocytes is 20 μg/ml of antigen (Figures 2 and 3) and the appropriate time is 120 and 72 hours, respectively.

**Figure 2. INF-γ response as a result of stimulation of mouse spleen lymphocytes with rAPN1, 2 weeks after the last injection in the tested mouse groups for optimization test.** Each column shows the average amount of IFN-γ induced in response to the stimulation dosage, at 120 hours after stimulation with APN1 protein. G3: group 3 of mice receiving 5 μg of APN1+ CFA/IFA antigen and C-: group of mice that did not receive any antigen (G7). The amount of antigen used to stimulate lymphocytes is shown below the columns. Con A was used as a positive control to stimulate lymphocytes.

**Figure 3- TNF-α response as a result of stimulation of mouse spleen lymphocytes with rAPN1, 2 weeks after the last injection in the tested mouse groups for optimization test.** Each of the columns shows the average amount of TNF-α induced in response to the stimulation dosage, at 72 hours after stimulation with APN1 protein. G3: group 3 of mice receiving 5 μg of APN1+ CFA/IFA antigen and C-: group of mice that did not receive any antigen. The amount of antigen used to stimulate lymphocytes is shown below the columns. Con A was used as a positive control to stimulate lymphocytes.
